# Hughes–Stovin syndrome as an uncommon cause of cardiac thrombus and pulmonary embolism: A case report

**DOI:** 10.1016/j.radcr.2026.02.031

**Published:** 2026-04-06

**Authors:** Ikram Tahani, Yasmine Ouaddouh, Taha Himri, Soumia Boulouiz, Zakaria Bazid, Noha El Ouafi, Nabila Ismaili

**Affiliations:** a Faculty of Medicine and Pharmacy, Moh I^st^ University, Oujda, Morocco; b Department of Cardiology, Mohammed VI University Hospital/Mohammed I University, Oujda, Morocco; c Laboratory of Epidemiology, Faculty of Medicine and Pharmacy, Clinical Research and Public Health, Oujda, Morocco

**Keywords:** Hughes–Stovin syndrome (HSS), Behçet’s disease (BD), Venous thromboembolism (VTE), Cardiac thrombi, Pulmonary artery anevrysm (PAAs)

## Abstract

Hughes–Stovin syndrome represents an exceptionally rare vasculitis disorder, with significant clinical convergence with Behçet’s disease. The syndrome is defined by the coexistence of venous thromboembolism and pulmonary artery anevrysms. Intracardiac thrombi although rare, predominates within the right sided cardiac chambers. We report the case of a 30-year-old male patient with a prior diagnosis of Behçet’s disease, presenting to emergency department with acute respiratory distress and echocardiographic signs of acute pulmonary heart and 2 large thrombi in the right ventricle, chest scan revealed bilateral pulmonary artery anevrysms along with alveolar hemorrhagic foci and recurrent pulmonary embolism. Through this case, we explore whether Hughes–Stovin syndrome represents a distinct clinical entity or a cardiovascular complication of Behçet's disease, and examine the therapeutic approaches associated with this emerging pattern.

## Introduction

Multiple predisposing factors have been described for venous VTE and cardiac thrombosis, including some that should not be neglected most notably HSS, a very rare disease, mostly affecting young men with common features shared with BD, with less than 90 cases reported until now [1]. While HSS is characterized by the coexistence of VTE with deep vein thrombosis occurring in the iliac and femoral veins, and pulmonary artery aneurysms, cardiac thrombi are especially rare, occurring in 8%-10 % in the right heart [2].

The clinical spectrum of HSS is predominantly characterized by recurrent episodes of deep venous thrombosis most frequently involving the lower limbs, together with the formation of pulmonary artery aneurysms [3,4]. The disease carries a particularly grave prognosis owing to the risk of catastrophic complications, including pulmonary hemorrhage and aneurysmal rupture [5].

In this case report, we present an uncommon case of HSS presenting with pulmonary arterial anevrysm and cardiac thrombi, in the context of a Behçet’s disease.

## Case presentation

A 30-year-old male patient with history of Behçet’s disease diagnosed based on recurrent uveitis, bipolar aphtosis, and multiple thrombotic events including a first episode of pulmonary embolism and deep vein thrombosis of femoral vein. Initial treatment with corticosteroids failed; and azathioprine (50 mg/day) was discontinued by the patient for financial reasons. Cyclophosphamide was proposed but refused due to concerns about infertility, the patient was then lost to follow up and wasn’t taking any treatment for BD.

The patient presented to our emergency department with New York Heart Association (NYHA) class II dyspnea, which he had been experiencing for the last 3 days, he also mentioned having asthenia with multiple episodes of hemoptysis. Upon clinical examination, the patient was conscious and tachypneic (23 breaths/min) with oxygen saturation of 89% on room air, improving to 95% with 3 L/min of oxygen, his blood pressure was at 121/61 mmHg, with a heart rate of 110 beats per minute.

Cardiovascular assessment demonstrated normal heart sounds, without evidence of murmurs, gallops, rubs, or clinical signs suggestive of heart failure. Pulmonary auscultation revealed preserved vesicular breath sounds, with no adventitious findings such as wheezes, rhonchi, or crackles. Multiple non-inflammatory inguinal lymphadenopathies measuring approximatively up to 2 centimeters were noted. The remainder of examination was normal.

**Fig. 1:**
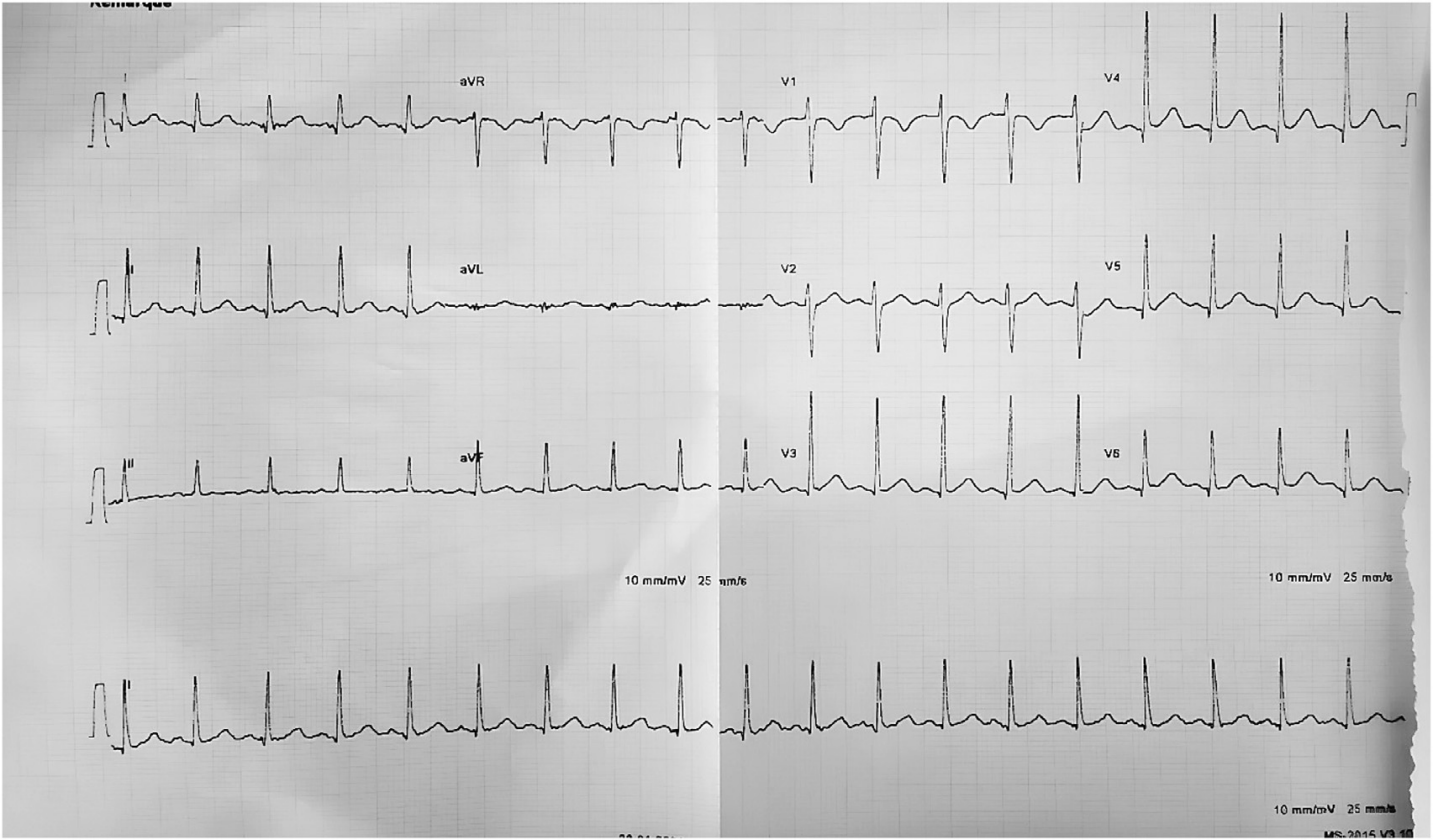
The 12-lead EKG showing a sinus tachycardia at 120 beats per minute and an incomplete right bundle brunch block.

The 12-lead electrocardiogram (EKG) revealed regular sinus rhythm with sinus tachycardia at 120 beats per minute and a new incomplete right bundle branch block (RBBB) (Fig. 1).

**Fig. 2:**
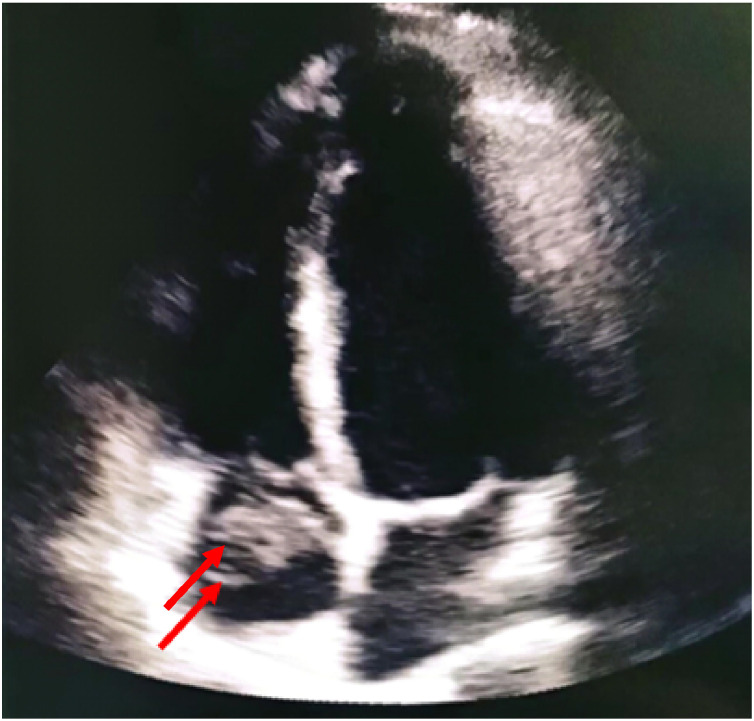
Apical 4-chamber transthoracic echocardiography showing a right atrial thrombus (red arrow).

An urgent Transthoracic echocardiography revealed a dilated right ventricle (RV/LV ratio = 1) with preserved systolic function. Tricuspid regurgitation allowed estimation of pulmonary artery systolic pressure at 50 mmHg using the simplified Bernoulli equation**.** Importantly, echocardiography revealed a well-defined intracavitary mass attached to the atrial wall, exhibiting homogenous echogenicity and limited vascularity. The mass displayed a smooth surface and relatively low mobility, extending into the atrial lumen without evidence of infiltration into adjacent cardiac structures. Doppler evaluation did not reveal internal blood flow within the lesion, which is consistent with avascular organized thrombus in the right atrium measuring 15 × 14 mm. In contrast, a neoplastic process was ruled out because it usually demonstrate heterogenous composition, with irregular contours and internal vascularity which was the case in our patient (Fig. 2).

Given the patient's known inflammatory thrombotic disease, a venous Doppler ultrasound was performed revealing an extensive deep vein thrombosis involving the right common femoral, superficial, and deep femoral veins.

**Fig. 3:**
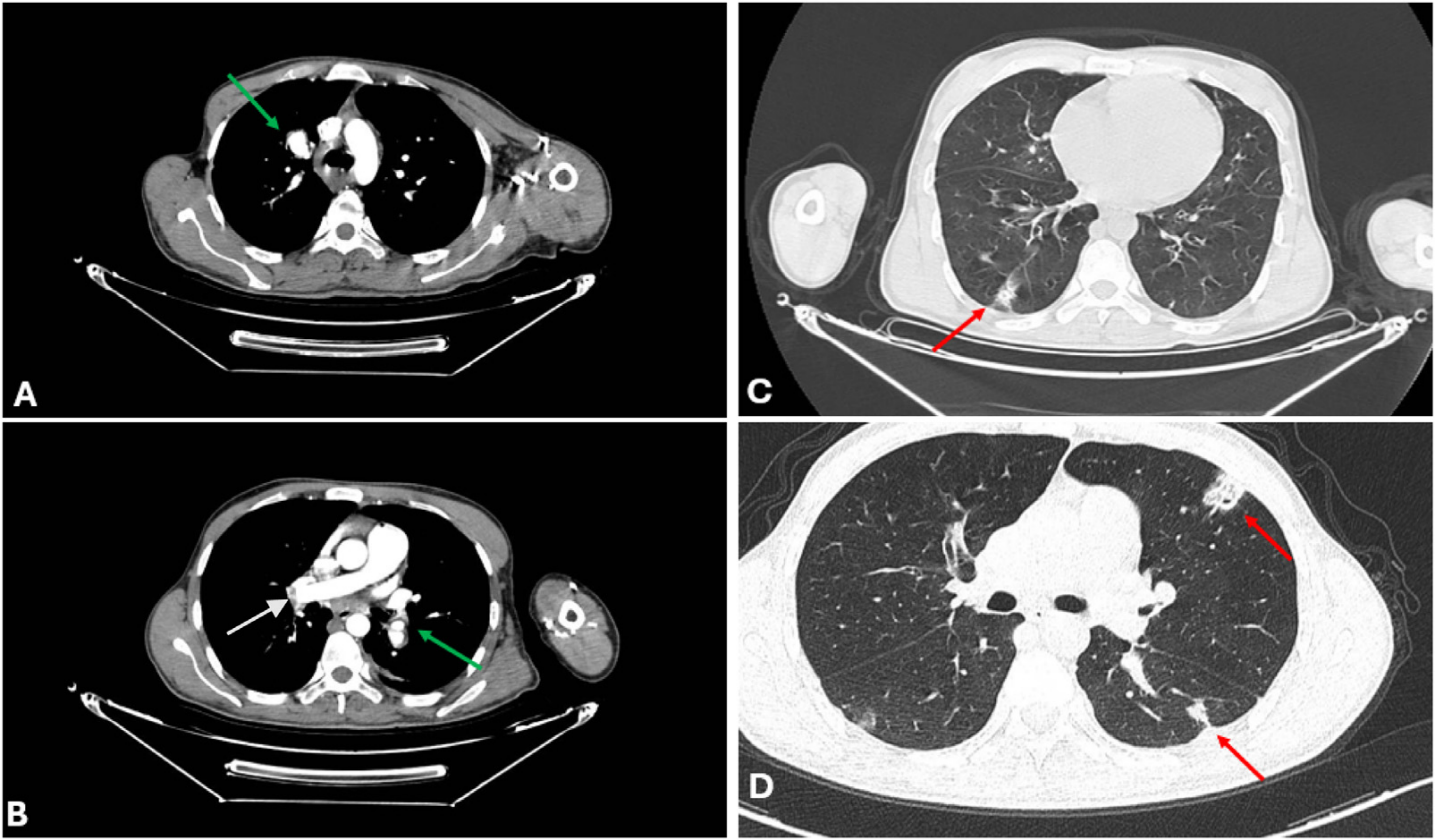
Chest angiography CT scan revealing pulmonary arterial anevrysms images A and B (green arrow), and hemorrhagic alveolar foci (red arrow) with pulmonary arterial embolism (white arrow).

Thoracic CT angiography revealed a new bilateral segmental pulmonary embolism, bilateral thrombosis of the inferior pulmonary veins, and saccular arterial aneurysms in the right apical segmental and left lower lobar branches. Additionally, hemorrhagic alveolar foci were observed in the right basal and left upper lobes, characterized by patchy, ill-defined, and ground-glass areas with peripheral distribution without evidence of volume loss or architectural distortion. These findings were consistent with alveolar filling from hemorrhage. In contrast, pulmonary infarcts typically manifest as wedge-shaped, pleural-based consolidations associated with vascular occlusions (Fig. 3).

Laboratory tests showed normal blood cell counts, and an elevated C-reactive protein at 0.015 g/dl, which is a marker of inflammation. Troponin levels were elevated at 1323 ng/mL (normal < 26 ng/mL), the proBNP test showed a high level, reaching a 1200 mg/L. Kidney and liver function were also normal, the rest of biological findings were also normal.

In our patient, coagulation laboratory investigations demonstrated negative anti-cardiolipin (aCL) and anti-β2 glycoprotein I (β2GPI) antibodies, no evidence of factor V Leiden or prothrombin gene mutations, and preserved levels of antithrombin III, protein C, and protein S were noted.

**Fig. 4:**
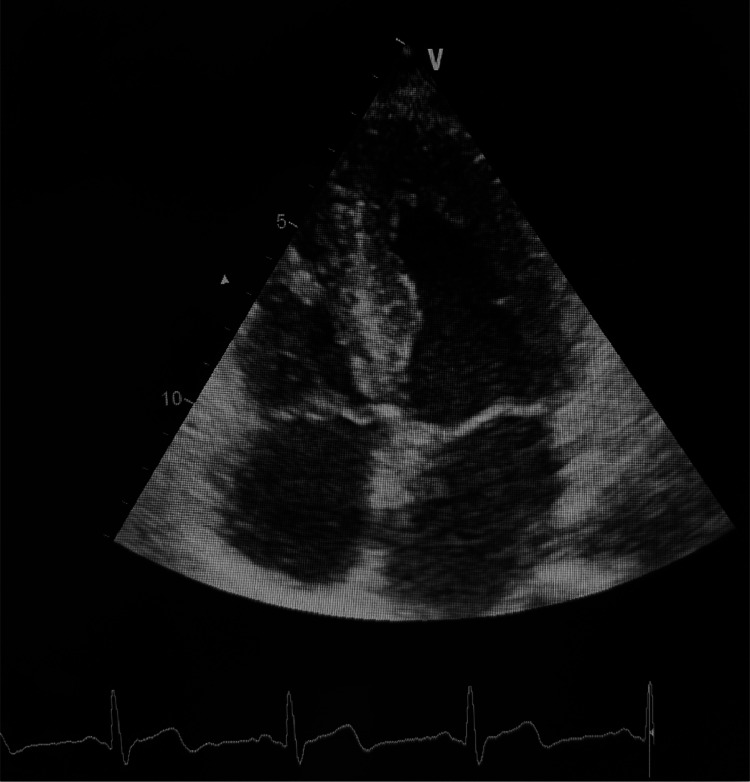
Three month follow up trans thoracic echocardiographic images of apical 4 chamber demonstrating clear resolution of the intra cardiac clot of the right atrium.

Based on the medical history of the patient, the clinical presentation and the CT scan imaging of venous thromboembolism and pulmonary arterial aneurysms and presence of intra cardiac thrombi, a diagnosis of Hughes–Stovin syndrome was made. The patient was then started on intravenous methylprednisolone (60 mg/day for 3 days), while Anticoagulation and antiplatelet therapy were discontinued due to alveolar hemorrhage, no thrombolytic procedures were undertaken. After 10 days of hospitalization and respiratory improvement, azathioprine therapy was initiated (Imurel 50 mg/day) which yielded a substantial clinical response and stabilization of pulmonary hemorrhage. The patient was discharged with vitamin K-antagonists: Acenocoumarol 3 mg per day, with close follow-up appointments. Echocardiography 3 months later confirmed resolution of the intra cardiac clot of the right atrium (Fig. 4). MRI was planned for the patient, but was not performed for financial limitations.

## Discussion

HSS is a rare and potentially life-threatening form of vasculitis, widely considered to represent a vascular variant within the spectrum of Behçet’s disease [2]. It is characterized by the combination of recurrent thrombophlebitis and pulmonary and/or bronchial artery anevrysm [2,3]. Globally, it mainly affects males (75,4 %) with a mean age of 33.8 years, and to our knowledge, fewer than 90 cases had been documented as of 2021, according to the Hughes–Stovin International Study (HSSISG) Group [6].

The disease typically progresses through 3 sequential phases: an initial stage characterized by thrombophlebitic manifestations, a subsequent stage marked by the development of pulmonary artery aneurysms (PAAs), and a final stage in which aneurysmal rupture precipitates massive hemoptysis, often with fatal outcomes [7].Clinical presentations are highly variable, with recurrent hemoptysis in nearly all affected individuals, dyspnea in 86% of cases, deep vein thrombosis in 33.3%, and superficial thrombophlebitis in 5.3% [6,7]. Neurological manifestations secondary to cerebral venous sinus thrombosis (CVST), including headache, diplopia, and seizures, are comparatively rare, as is intracardiac thrombosis, which was observed in 12 patients (21.1%) within the HSSISG cohort [6].

The HSSISG, representing the largest cohort studied to date (57 cases), proposed 3 principal diagnostic criteria: (a) diffuse vasculo-occlusive manifestations, including recurrent superficial thrombophlebitis, deep vein thrombosis (DVT), cerebral venous sinus thrombosis (CVST), intracardiac thrombosis, or arterial thrombosis; (b) normal coagulation parameters, encompassing negative anti-cardiolipin (aCL) and anti-β2 glycoprotein I (β2GPI) antibodies, absence of factor V Leiden and prothrombin gene mutations, and no deficiencies in antithrombin III, protein C, or protein S; and (c) radiological evidence on computed tomography pulmonary angiography (CTPA) of pulmonary artery aneurysms (PAAs), with or without adherent intra-aneurysmal thrombus or aneurysmal wall enhancement on post-contrast imaging [6].

Therapeutic strategies for Hughes–Stovin syndrome (HSS) have primarily centered on corticosteroids, either as monotherapy or in combination with systemic immunosuppressive agents, including cytotoxic regimens such as cyclophosphamide with glucocorticoids. Evidence suggests that in patients with HSS, the administration of immunosuppressive therapy—particularly in the absence of, or with only minimal, hemoptysis—may contribute to the stabilization of pulmonary artery aneurysm walls and, in some cases, lead to their complete resolution [7,8].

The management of pulmonary embolism (PE) in the context of Hughes–Stovin syndrome (HSS) presents a significant therapeutic dilemma, particularly when concomitant pulmonary artery aneurysms (PAAs) and hemoptysis are present [9]. Although anticoagulation may limit thromboembolic progression, its use is generally contraindicated due to the heightened risk of catastrophic hemorrhage [7]. In cases complicated by right ventricular thrombus (RVT), urgent thrombolysis or surgical embolectomy may be warranted for type A thrombi, given their high embolic potential and associated mortality, whereas type B thrombi carry a more favorable prognosis and are not typically managed with thrombolysis [10]. Surgical resection has been attempted in select cases with localized high-risk aneurysms; however, the multifocal and bilateral nature of PAAs in most patients with HSS, coupled with the considerable morbidity and mortality of surgery, restricts its role as a primary modality [6]. In such scenarios, transcatheter embolization has emerged as a safer and more effective life-saving intervention, including bronchial artery embolization when bronchial artery aneurysms are detected [7].

In our patient, the therapeutic strategy prioritized stabilization of aneurysmal walls and mitigation of rebleeding risk; therefore, anticoagulation and invasive interventions were avoided in favor of initial pulse therapy with methylprednisolone combined with infliximab. This case underscores the critical need to systematically screen patients with Behçet’s disease for Hughes–Stovin syndrome, particularly through the detection of pulmonary and bronchial arterial anomalies, and further emphasizes the role of early embolization—especially of leaking pulmonary artery aneurysms with compromised wall integrity—to prevent rupture and fatal hemoptysis [8].

Imaging also plays a central role in the longitudinal surveillance and management of HSS cases. Serial chest CT or MRI allows non-invasive monitoring of parenchymal and vascular changes over time, enabling clinicians to detect disease progression, complications, or treatment response at an early stage. In particular, high-resolution modalities facilitate assessment of evolving consolidation, hemorrhage, vascular remodeling, or fibrotic transformation, which may not be clinically apparent in the early phases [7]. These imaging data can guide therapeutic decisions, such as the need to escalate immunomodulatory therapy, adjust anticoagulation strategies, or pursue interventional procedures, thereby contributing to individualized patient management [8]. Overall, systematic imaging surveillance provides an indispensable adjunct to clinical and laboratory evaluation, enhancing diagnostic confidence and optimizing outcomes in patients with HSS [8].

## Conclusion

Our case highlights the importance of considering Hughes–Stovin syndrome in Behçet’s disease with atypical thrombotic or aneurysmal involvement, especially of the right heart. Clinicians should maintain a high index of suspicion in patients with unexplained thromboembolic events, particularly in the presence of pulmonary artery aneurysms, where early detection and timely intervention may be lifesaving, in this exceptionally rare yet life-threatening vasculitic disorder.

## Patient consent

Written informed consent was obtained from the patient for publication of this case report and accompanying images.
